# Supplementary figures and images for: Genetic relationship and source species identification of 58 Qi-Nan germplasms of Aquilaria species in China that easily form agarwood
Source: PLoS One. 2022 Jun 16;17(6):e0270167. doi: 10.1371/journal.pone.0270167 (PMC9202955; doi:10.1371/journal.pone.0270167)

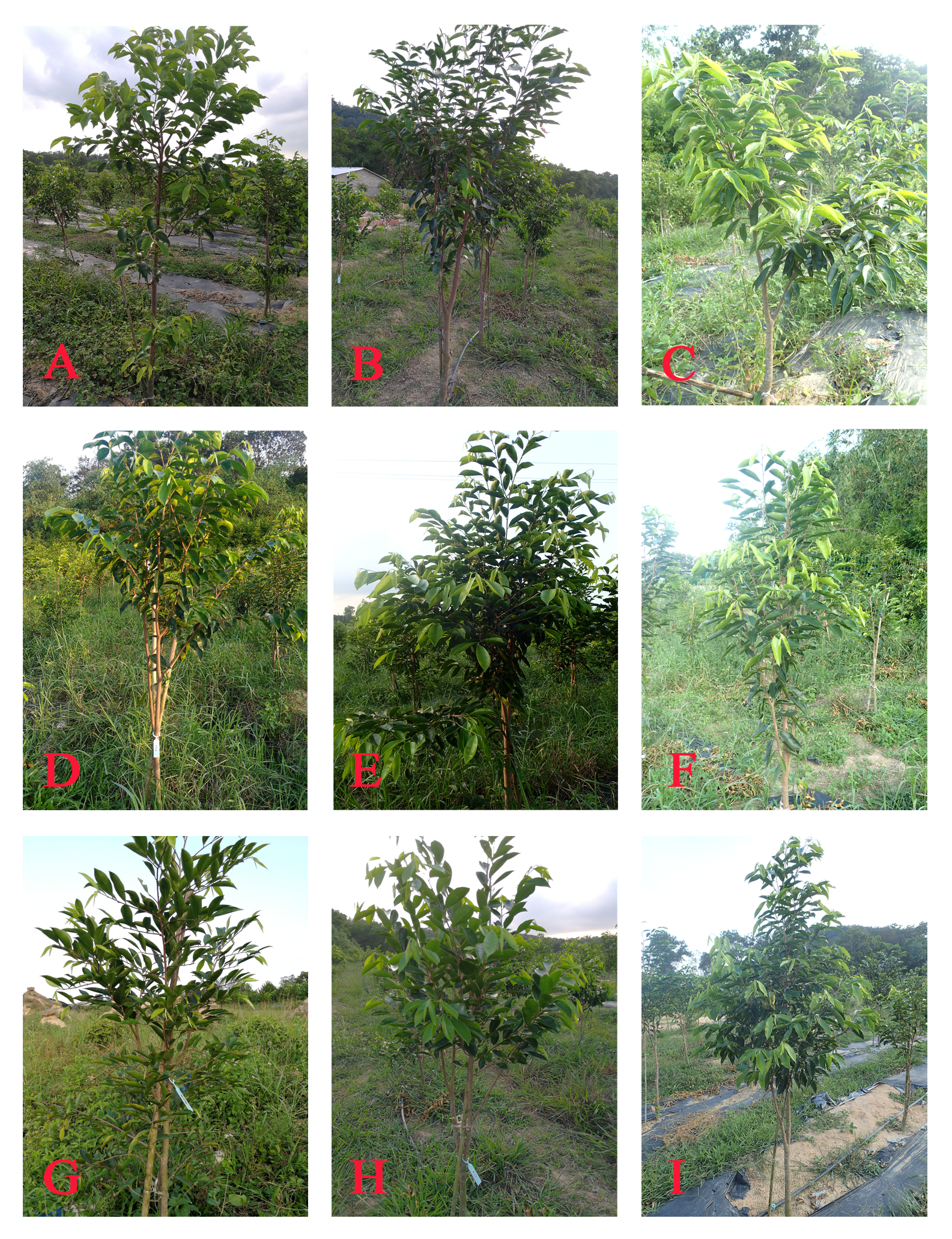

Supplement: S1 Fig — A: CN-TJ-DA. B: CN-QLX-DB. C: CN-JSY-DB. D: CN-DYP-DB. E: CN-LBS-DB. F: CN-LYW-DB. G: CN-ZTJ-DB. H: CN-ZSZ-DB. I: CN-XS3HCQ-XL. (TIF) [file pone.0270167.s001.tif]
