# Supplementary material for: Genetic relationship and source species identification of 58 Qi-Nan germplasms of Aquilaria species in China that easily form agarwood
Source: PLoS One. 2022 Jun 16;17(6):e0270167. doi: 10.1371/journal.pone.0270167 (PMC9202955; doi:10.1371/journal.pone.0270167)
Supplement: Attachment — Submitted filename: Response letter.docx [file pone.0270167.s005.docx]

Dear Dr. Pankaj Bhardwaj,

Academic Editor, PLOS ONE

We would like to thank you and the two reviewers for your careful and thoughtful consideration of our manuscript. In particular, we were pleased that the Editor and reviewers were interested in the identification of Qi-Nan germplasms. We have taken care to integrate the suggestions brought up during review and feel that the paper is greatly improved by the process. We are confident that we have addressed the concerns brought up during review.

Sincerely,

Yong Kang

**Comment is from Reviewer 1**

**Comment:** The authors have attempted to identify germplasm resources of *Aquilaria species* using DNA barcoding analysis with the help of one nuclear and chloroplast genes or their combination. The manuscript is well written and the results are clearly presented. However, to get a better resolution of genetic differences, it is highly recommended to use molecular markers like SSR or ISSR or a combination of DNA barcoding and these molecular markers.

**Response:** Thank you very much for your advice. DNA barcoding technology has been widely used in species identification, especially in the identification of closely related species and germplasm resources. It has the advantages of strong objectivity, high accuracy and rapid identification. The combinations ITS2+*matK* had the highest success rate, the correct match rates of “best match”, “best close match” and “all species” for these combinations were 93.84% in this study. The results can support the accuracy of identification of the source species of Qi-Nan germplasms. However, I will focus on the combination of SSR or ISSR with DNA barcoding to get a better resolution of genetic differences in the future research.

**Comments are from Reviewer 2**

“Qi-Nan” is an important agarwood species valued for its fragrance and medicinal importance. Authors have made efforts to identify its source species using DNA barcoding analysis and inferred *A. sinensis.* to be the source species of “Qi-Nan”. The overall quality of manuscript the way it is written needs to be improved.

The major concerns of reviewers are as below:

**Comment 1:** Please mark Figure 1 properly.

**Response:** Thanks for your suggestion. Figure 1 has been marked.

**Comment 2:** In line 136, please provide the full detail of the DNA isolation kit used.

**Response:** Thank you for your comments. Full details of the DNA isolation kit used have been supplemented.

**Comment** **3:** It will be appropriate the address the primer pairs used in this study as universal primers rather than common primers (LINE 136).

**Response:** Thanks very much. I have finished the modification according to your comments.

**Comment** **4:** Please rewrite methods sections, some of the information is duplicated while some sections need elaboration e.q.1) Data analysis section contain the duplicated information. 2) the name of sequencing platform and the methodology of sequencing is not provided.

**Response:** Thank you very much for your review. I have rewritten the method section. On the one hand, the content of duplicate information in data analysis has been modified. And on the other hand, DNA sequencing methods and platforms have been supplemented.

**Comment** **5:** Reference 2 is not complete.

**Response:** Thanks for your suggestion. I have replaced reference 2.

**Comment** **6:** In general, few references are misleading in the manuscript. Authors are requested to recheck the citation used in the manuscript.

**Response:** Thank you for your valuable comments. I have rechecked and revised the citations used in the manuscript.

**Comment** **7:** It would have been appropriate to dedicate the last section of discussions to conclusion and future aspects but the last section appears more of the introductory part of the manuscript.

**Response:** Thank you very much for your advice. I have revised in the discussion and conclusion according to your comments.
